# Tailoring of Selenium-Plated Novasomes for Fine-Tuning Pharmacokinetic and Tumor Uptake of Quercetin: In Vitro Optimization and In Vivo Radiobiodistribution Assessment in Ehrlich Tumor-Bearing Mice

**DOI:** 10.3390/pharmaceutics14040875

**Published:** 2022-04-16

**Authors:** Heba M. Aboud, Amal K. Hussein, Abdallah Z. Zayan, Tarek Saad Makram, Mona O. Sarhan, Dina M. El-Sharawy

**Affiliations:** 1 Department of Pharmaceutics and Industrial Pharmacy, Faculty of Pharmacy, Beni-Suef University, Beni-Suef 62514, Egypt; 2 Department of Pharmaceutics, Faculty of Pharmacy, Minia University, Minia 61519, Egypt; amal.hussein@mu.edu.eg; 3 Department of Pharmaceutics, Faculty of Pharmacy, Nahda University, Beni-Suef 62513, Egypt; abdalah.zidan@nub.edu.eg; 4 Department of Pharmaceutics and Industrial Pharmacy, Faculty of Pharmacy, October 6 University, Giza 12585, Egypt; tareksaadmakram@yahoo.com; 5 Labeled Compounds Department, Hot Labs Center, Egyptian Atomic Energy Authority (EAEA), Cairo 13759, Egypt; monasarhan@windowslive.com (M.O.S.); tinaa81@hotmail.com (D.M.E.-S.); 6 Cyclotron Project, Nuclear Research Center, Egyptian Atomic Energy Authority (EAEA), Cairo 13759, Egypt

**Keywords:** quercetin, novasomes, selenium, tumor targeting, in vivo biodistribution, radiolabeling

## Abstract

Quercetin (QRC) is a bioflavonoid with anti-inflammatory, antioxidant, and anticancer activities, yet QRC poor bioavailability has hampered its clinical implementation. The aim of the current work was to harness novasomes (NOVs), free fatty acid enriched vesicles, as a novel nano-cargo for felicitous QRC delivery with subsequent functionalization with selenium (SeNOVs), to extend the systemic bio-fate of NOVs and potentiate QRC anticancer efficacy through the synergy with selenium. QRC-NOVs were primed embedding oleic acid, Brij 35, and cholesterol adopting thin-film hydration technique according to Box–Behnken design. Employing Design-Expert^®^ software, the impact of formulation variables on NOVs physicochemical characteristics besides the optimum formulation election were explored. Based on the optimal NOVs formulation, QRC-SeNOVs were assembled via electrostatic complexation/in situ reduction method. The MTT cytotoxicity assay of the uncoated, and coated nanovectors versus crude QRC was investigated in human rhabdomyosarcoma (RD) cells. The in vivo pharmacokinetic and biodistribution studies after intravenous administrations of technetium-99m (^99m^Tc)-labeled QRC-NOVs, QRC-SeNOVs, and QRC-solution were scrutinized in Ehrlich tumor-bearing mice. QRC-NOVs and QRC-SeNOVs disclosed entrapment efficiency of 67.21 and 70.85%, vesicle size of 107.29 and 129.16 nm, ζ potential of −34.71 and −43.25 mV, and accumulatively released 43.26 and 31.30% QRC within 24 h, respectively. Additionally, QRC-SeNOVs manifested a far lower IC_50_ of 5.56 μg/mL on RD cells than that of QRC-NOVs (17.63 μg/mL) and crude QRC (38.71 μg/mL). Moreover, the biodistribution study elicited higher preferential uptake of ^99m^Tc-QRC-SeNOVs within the tumorous tissues by 1.73- and 5.67-fold as compared to ^99m^Tc-QRC-NOVs and ^99m^Tc-QRC-solution, respectively. Furthermore, the relative uptake efficiency of ^99m^Tc-QRC-SeNOVs was 5.78, the concentration efficiency was 4.74 and the drug-targeting efficiency was 3.21. Hence, the engineered QRC-SeNOVs could confer an auspicious hybrid nanoparadigm for QRC delivery with fine-tuned pharmacokinetics, and synergized antitumor traits.

## 1. Introduction

Flavonoids constitute an immense group of naturally occurring polyphenols, which possess ubiquitous dispersal throughout the plant kingdom, hence existing in commonly human-ingested fruits, vegetables, and nuts. Amongst flavonoids, quercetin (QRC, 3,3′,4′,5,7-pentahydroxyflavone) represents the milestone compound within the subclass of flavonol. QRC exerts diverse biological and clinical actions, including antioxidation, anti-inflammation, anti-anemia, anti-anaphylaxis, coronary arteries dilatation, antihyperlipidemia, anti-platelet aggregation, anti-diabetes, neuroprotection, and anticancer impacts [1]. Currently, the prophylactic and therapeutic activities of QRC for cancer tackling have triggered curiosity. Numerous studies have braced the aptitude of QRC to suppress cellular proliferation of multiple cancers such as pancreatic, prostatic, lung, as well as colon tumors, besides inhibition of angiogenesis and metastasis, in addition to evoking apoptosis of cancerous cells at micromolar levels [2]. Moreover, QRC can abrogate multi-drug resistance in tumor cells and promote the antitumor influences of other drug modalities [3].

Despite such remarkable pharmacological traits, QRC in vivo exploitation is overtly hampered by its sparse aqueous solubility, diminished gastrointestinal tract absorption, limited physiological milieu stability, poor permeability, extensive first-pass metabolism, and short biological half-life [4]. Notably, QRC oral bioavailability is lower than 17% in rats [5] and approximately 1% in humans [6]. Addressing the aforementioned encumbrances is spotlighted as a key task upon tailoring formulations for clinical application. Thus, it is crucial to ameliorate QRC bioavailability by employing novel strategies.

Intriguingly, different delivery systems were proposed for boosting QRC bioavailability based on polymeric mixed micelles [7], nanodiamond [8], zein nanoparticles [9], and poly(D,L-lactide-co-glycolide) (PLGA) nanoparticles [10] where its antitumorigenic, antiproliferative, anti-inflammatory, and antioxidant effects, respectively were augmented. Herein, novasomes (NOVs), non-phospholipid vesicles, were refined as a novel system for consolidating the bioavailability of QRC.

NOVs were primarily created as a patented approach by Novavax, IGI laboratories to triumph over associated troubles with current vesicular delivery systems. NOVs are the modulated paragons of liposomes or niosomes assembled from the blend of free fatty acids and the polyoxyethylene fatty acids monoester and cholesterol [11]. NOVs can also be described as phospholipid-devoid paucilamellar vesicles of about 0.1 to 1.0 µm diameter. NOVs possess appealing aspects such as being multi-bilayered vesicles with a central core of high amplitude in appropriate size range, as well as they can transport high concentrations of the laden drugs [12]. Based on NOVs, many vaccines have been licensed [13]. The implementation of NOVs for accentuating brain targeting of zolmitriptan via trans-nasal delivery [14], as well as topical delivery of terconazole [15] was previously reported. Only, the current study investigates the potential of this novel nano-cargo for QRC tumor targeting.

NOVs, free fatty acid-enriched vesicles, are anticipated to have greater diffusion across the biological membranes and hasten the tumor uptake of the laded drug. Also, NOVs can exert passive accumulation in the tumorous tissues through the enhanced permeability and retention (EPR) impact, though they may be succumbed to fast elimination as a result of complement conjugation and phagocytosis via the reticuloendothelial system (RES) [16,17]. The small circulatory half-life of common vesicles mitigates the EPR influence; thus, extensive effort is mandatory for enhancing the in vivo bio-fate of such conventional vesicles. In this perspective, we hypothesized that selenium-plated NOVs (SeNOVs) can be suggested as an auspicious strategy to upgrading the architecture of NOVs, strengthening their plasma stability, as well as potentiating the anticancer effect of QRC.

Selenium is a vital trace element, which serves as a catalytic component of some antioxidant enzymes such as thioredoxin reductase, and glutathione peroxidases. It has a pivotal role in normal body growth and life preservation. The unique biological functions of selenium in tumor chemotherapy and chemoprevention have been verified [18]. Selenium nanoparticles as drug delivery nano-cargo and anticancer domain have been iteratively assembled and appraised [19,20]. Due to their greater density, selenium nanoparticles can be embedded into cancer cells via endocytosis, hence triggering cell death by inducing mitochondrial-primed apoptosis [21]. To the best of our knowledge, the synergy of selenium and QRC-NOVs for tumor burden intervention has not been yet explored.

The aim of this work was to investigate the potential of NOVs for systemic delivery of QRC via hybrid platform based on selenium for tuning QRC pharmacokinetics and tumor targeting. Adopting Box–Behnken design, the influences of NOVs formulation variables on drug entrapment, vesicle size, as well as the in vitro release were scrutinized. Then, QRC-SeNOVs were assembled through electrostatic complexation/in situ reduction method based on the optimum NOVs formulation, and both of them were tested against human rhabdomyosarcoma (RD) cells versus crude QRC. Furthermore, QRC was radiolabeled utilizing technetium-99m (^99m^Tc) to assess its pharmacokinetics and biodistribution after intravenous (i.v.) administration of the tailored ^99m^Tc-QRC-SeNOVs to Ehrlich tumor-bearing mice and compared to i.v. ^99m^Tc-QRC-NOVs and i.v. ^99m^Tc-QRC solution.

## 2. Materials and Methods

### 2.1. Materials

Quercetin, cholesterol, Brij 35 (polyoxyethylene lauryl ether), Tween 80 (polyoxyethylene sorbitan monooleate), sodium selenite, ascorbic acid, methylene chloride (HPLC grade), absolute ethanol (HPLC grade), and glacial acetic acid (HPLC grade) were procured from Sigma-Aldrich (St. Louis, MO, USA). Dialysis bags (Mol. Wt. cut off = 12,000 Da) were acquired from SERVA Electrophoresis GmbH (Heidelberg, Germany). Oleic acid, polyethylene glycol 400, potassium dihydrogen orthophosphate, potassium chloride, disodium hydrogen orthophosphate, and sodium chloride were provided by El-Nasr pharmaceutical chemical company (Cairo, Egypt). Technetium-99m was eluted as ^99m^TcO_4_^−^ from ^99^Mo/^99m^Tc generator, Radioisotope Production Facility, Cairo, Egypt. All other chemicals and solvents were of analytical grade and were used as received without any further modifications.

### 2.2. Experimental Design

A three-level three-factor Box–Behnken design was utilized for statistical optimization of the formulation variables for engineering of QRC-NOVs so as to attain optimum entrapment efficiency, smallest vesicle size, and highest QRC release. The experimental design was generated and evaluated by applying Design Expert^®^ software (version 12.0.3.0, Stat-Ease Inc. Minneapolis, MN, USA). A total of 15 experimental runs were established; 12 of which denote the mid-point of each edge of the multidimensional cube whilst the rest three represent the replicates of the cube’s center point. The three scrutinized causal factors were the concentrations of free fatty acid (oleic acid) (*X*_1_), surfactant (Brij 35) (*X*_2_), and cholesterol (*X*_3_). The entrapment efficiency percent (*Y*_1_: EE%), vesicle size (*Y*_2_) and accumulative % QRC released from NOVs over 24 h (*Y*_3_: Q_24h_) were picked as the dependent variables. The independent (low, medium, and high levels) as well as dependent variables are depicted in Table 1. The composition of the tailored QRC-NOVs according to Box–Behnken design is outlined in Table 2.

### 2.3. Fabrication of QRC-NOVs

QRC-NOVs were elaborated adopting thin film hydration technique with a minor modification [14]. Briefly, an amount of 10 mg QRC along with specific weights of oleic acid, Brij 35, and cholesterol were dissolved in 10 mL mixed organic solvents of methylene chloride, and ethanol (2:1 *v*/*v*). Utilizing a rotary evaporator (Heidolph Laborota 4000 Series, Heizbad, Germany) at 60 rpm, the organic solvents were steadily evaporated at 60 °C under vacuum, whereby a thin dry film on the walls of the round bottom flask was formed. Afterwards, hydration of the nascent film with 10 mL phosphate buffer saline (PBS) pH 7.4 was executed via rotating the flask within a water bath at 60 °C for 30 min under ordinary pressure. Sonication of the resultant vesicles was performed for 10 min in a bath sonicator (Sonix TV ss-series, North Charleston, SC, USA) to lessen the vesicles size [22]. The assembled nanodispersions were chilled overnight for maturation at 4 °C.

### 2.4. In Vitro Characterization of QRC-NOVs

#### 2.4.1. Determination of QRC Entrapment Efficiency Percent (EE%)

QRC-NOVs were secluded from the unentrapped QRC by centrifugation of the nanodispersions at 15,000 rpm for 2 h at 4 °C (Sigma Laborzentrifugen, Osterode am Harz, Germany). The yielded residue was washed twice with 5 mL distilled water and recentrifuged to warrant complete separation of the unentrapped drug from the voids between the vesicles [23,24]. The detached NOVs were disrupted utilizing 5 mL absolute ethanol, and vortex-mixed to confirm total lysis of the vesicles. Further, the mixture was recentrifuged for 30 min at 10,000 rpm for sedimentation of any debris. At the end, the quantity of entrapped QRC was assayed, in triplicate, spectrophotometrically (Shimadzu UV-1800, Tokyo, Japan) at λ_max_ 375 nm after proper dilution of the clear supernatant with PBS pH 7.4. The EE% of QRC was computed according to the following equation:

(1) $$EE\%=\frac{Amount\;of\;QRC\;entrapped}{Total\;amount\;of\;QRC}\times 100\;$$

#### 2.4.2. Determination of Vesicle Size and ζ Potential

The average vesicle size, polydispersity index (PDI), and ζ potential of QRC-NOVs were estimated by dynamic light scattering (DLS) (Zetasizer Nano ZS, Malvern instruments, Malvern, UK). Dilution of the samples with PBS pH 7.4 was performed before assessment to possess an appropriate scattering intensity. All measurements were carried out in triplicate at a temperature of 25 ± 2 °C and an angle of 90° to the incident beam [25], and the mean values ± SD procured were recorded.

#### 2.4.3. In Vitro Release Study of QRC-NOVs

The membrane diffusion procedure [26] was adopted for estimation of QRC release, in triplicate, from the assembled NOVs utilizing an Erweka DT-720 USP dissolution tester, type 1 (Heusenstamm, Germany). Based on the computed EE%, precise aliquots of QRC-NOVs (correspondent to 2 mg of QRC) were inserted into glass cylinders (2.5 cm internal diameter and 6 cm length) rigorously concealed from one end with the presoaked dialysis membrane (Mol. Wt. cut off = 12,000 Da). The loaded glass cylinders were moored at the shafts of the USP dissolution tester. To ascertain sink condition, 200 mL of PBS pH 7.4 comprising 0.01% *v*/*v* Tween 80 was used as a release milieu [27]. Customization of the rotation speed at 100 rpm with temperature set to 37 ± 0.5 °C during the release experimentation was executed. Aliquots (1 mL) were pulled regularly at scheduled time intervals of 1, 2, 4, 6, 8, 16, and finally at 24 h, then, they were recharged with fresh milieu of equivalent volume to warrant fixed volume. Following samples filtration via 0.45 μm membrane filter, spectrophotometric measurement at λ_max_ 375 nm was performed for QRC content. The accumulative % QRC-NOVs released was graphed versus time. Also, the in vitro release pattern of 1 mL of free QRC solution (2 mg/mL in PBS pH 7.4 containing 60% *v*/*v* polyethylene glycol 400) was similarly investigated. For assessment of the release kinetics of QRC-NOVs, the compiled data were fitted into zero-order, first-order, Higuchi, Hixson–Crowell, and Korsmeyer–Peppas models. Considering the magnitude of determination coefficients (R^2^), the appropriate arithmetic model was picked.

### 2.5. Optimization of QRC-NOVs

The optimum formulation was refined applying the Design Expert^®^ software via harnessing constraints on the EE%, and Q_24h_ of QRC-NOVs to accomplish the maximal values, as well as on vesicle size to acquire the minimal value based on the desirability criterion. The solution with a desirability index close to 1 was elected. The proposed optimal formulation was then fabricated and scrutinized in triplicate for inspection of the validity of the computed optimized formulation causal factors, and prognosticated dependent responses provided by the software (*n* = 3).

### 2.6. Formulation of QRC-SeNOVs

To fabricate QRC-SeNOVs, a slightly modified electrostatic complexation/in situ reduction method as reported by Yin et al. [28] was implemented, where sodium selenite, and ascorbic acid were felicitously embedded into the optimum QRC-NOVs dispersion. In particular, accurately weighed amounts of sodium selenite, and ascorbic acid (dissolved in 1% *v*/*v* glacial acetic acid solution) at a molar ratio of 1:5 were introduced into QRC-NOVs suspension with vigorous stirring at 600–800 rpm on a magnetic stirrer for 6 h at ambient temperature. The color of the mixture shifted to orange-red as the reaction proceeded eliciting the development of QRC-SeNOVs since Se^4+^ was reduced to Se with concomitant precipitation on the surface of QRC-NOVs. Finally, the residual unreacted moieties were separated by dialysis against acetic acid solution (1% *v*/*v*) for 12 h [29]. The employed quantity of sodium selenite in the system was optimized to acquire a preferable formulation. To verify the formation of QRC-SeNOVs, optical absorption spectra through ultraviolet-visible (UV-Vis) spectrophotometry at a scanning range of 200–400 nm besides Fourier transform-infrared spectroscopy (FT-IR), as will be mentioned later, were performed [30]. Also, the previously described procedures for QRC-NOVs characterization with respect to EE%, vesicle size, ζ potential, and in vitro drug release were similarly adopted.

### 2.7. FT-IR Analysis

FT-IR spectra of various specimens were recorded on an FT-IR spectrometer (IR435-U-04, Shimadzu, Kyoto, Japan) with an attenuated total reflectance cell in the range of 4000–400 cm^−1^, resolution 4 cm^−1^. For each specimen, three replicates were attained, and spectra were further processed via the provided software with the instrument.

### 2.8. Transmission Electron Microscopy (TEM)

The morphological features of the optimal QRC-NOVs, and QRC-SeNOVs were explored via transmission electron microscope (JEM-1400, Jeol, Tokyo, Japan). A drop from each dispersion was spotted on a copper grid, and the excess was rubbed exploiting a filter paper. Next, a drop of aqueous phosphotungstic acid solution (2% *w*/*v*, negative staining) was introduced, and the excess was removed in a similar manner. Finally, examination of air-dried samples was executed utilizing TEM at 80 kV [31].

### 2.9. Physical Stability Study

To inspect the physical stability of the optimum QRC-NOVs, and QRC-SeNOVs, both formulations were stored for three months in glass vials at refrigeration temperature (4 ± 1 °C). The characteristics of the formulations were examined at predetermined intervals (0, 30, 60, and 90 days). Samples from each formulation were withdrawn, and then estimated for their physical appearance, EE%, vesicle size, and ζ potential. The analyses were performed in triplicate, and the mean values ± SD were narrated [23].

### 2.10. Cytotoxicity Evaluation Utilizing MTT Viability Assay

RD cell lines were procured from the American Type Culture Collection (ATCC, Rockville, MD, USA). RPMI-1640 milieu, supplied with gentamycin (50 µg/mL) and inactivated fetal calf serum (10%), was used for the cells’ growth. RD cell lines were subcultured twice to thrice weekly and kept in a moistened atmosphere with 5% CO_2_ at 37 °C. For antitumor assessment, RD cell lines were dispersed in the milieu at 5 × 10^4^ cell/well concentration in Corning^®^ 96-well tissue culture plates supervened by incubation for 24 h. Thereafter, the inspected formulations (free QRC, QRC-NOVs or QRC-SeNOVs) were incorporated into 96-well plates (*n* = 3) to accomplish 12 levels for every formulation. A 0.5% DMSO or milieus were employed as six vehicle controls and run for each 96-well plate. Following incubation for 48 h, the MTT test was utilized for estimation of the viable cells number. In brief, the milieus were isolated from the 96-well plate, then substituted with fresh RPMI-1640 culture milieu (100 µL) devoid of phenol red and 10 µL of the 12 mM MTT stock solution (5 mg MTT/mL in PBS pH 7.4) was added to every well involving the plain controls. Next, incubation of the 96-well plates at 5% CO_2_ and 37 °C for 4 h was carried out. An aliquot of the milieus (85 µL) was detached from the wells and 0.5% DMSO (50 µL) was introduced to every well, thoroughly mixed via the pipette and incubated for 10 min at 37 °C. Afterwards, the optical density was recorded at 590 nm through the microplate reader (SunRise, TECAN, Inc, Salzburg, Austria) for counting the viable cells number and the % viability was computed as follows:

(2) $$\%\;viability=\frac{OD_{t}}{OD_{c}}\times 100\;$$

where *OD_t_* is the average optical density of the tested sample-treated wells whilst *OD_c_* is the average optical density of control cells. The correlation amongst the surviving cells and drug level is graphed to attain the survival curve of every tumor cell line following treatment with the explored formulations. The IC_50_, the concentration warranted to trigger toxic influences in 50% of intact cells, was determined from graphical plots of the dose-response curve for each concentration utilizing Graphpad Prism software version 6.01 (San Diego, CA, USA) [32].

### 2.11. Biodistribution and Pharmacokinetic Studies Using Radiolabeling Technique

#### 2.11.1. Radiolabeling of QRC, QRC-NOVs and QRC-SeNOVs

QRC solution, QRC-NOVs, and QRC-SeNOVs were radiolabeled by technetium-99m (^99m^Tc) adopting the simple reducing method utilizing sodium dithionite [14]. The impact of sodium dithionite amount (0.5–20 mg), QRC amount (QRC solution or QRC nanodispersions equivalent to 0.25–2 mg), and incubation time (0.08–24 h) on radiolabeling efficacy was investigated to accomplish the optimal reaction conditions, then approximately 100 μL of the ^99^Mo/^99m^Tc generator eluate comprising 7.2 MBq of ^99m^TcO4^−^ was introduced each time. Only one factor was varied while the other factors were held constant, where each experiment was repeated thrice, and the results were reported as mean ± SD. The optimum radiolabeled-drug complex was inspected for in vitro stability.

##### Determination of the Radiolabeling Yield (RLY)

The influence of various labeling conditions on the RLY was scrutinized applying paper chromatography (utilizing strips of Whatman paper no. 1). Dual solvent systems were employed as mobile phases for assessment of the RLY. Acetone was utilized as a mobile phase for estimation of free ^99m^TcO_4_^−^ percentage, whereas the ^99m^Tc-colloidal impurities were appraised through using 0.5 M NaOH [33].

#### 2.11.2. Drug Biodistribution and Pharmacokinetic Studies

##### Animals

Swiss male albino mice (22–25 g), purchased from National Cancer Institute (Cairo, Egypt), were enrolled in this experiments. The mice were supplied with a pellet diet and tap water *ad libitum*. They were arbitrarily distributed in polyacrylic cages and remained under standardized laboratory circumstances of 12/12 h dark/light cycle at ambient temperature (25 ± 2 °C) and relative humidity of 60 ± 5%. Treatment of the animals was performed according to the approval of research ethics committee in National Cancer Research and Technology–Egyptian Atomic Energy Authority, Cairo, Egypt (approval no. 35A/21). The animal studies conform to the National Institutes of Health Guide for the Care and Use of Laboratory Animals (NIH Publications No. 8023, revised 1978).

##### Induction of Ehrlich Solid Tumor in Mice

Under aseptic circumstances, Ehrlich ascites carcinoma (EAC) was isolated from the ascetic exudate of a mouse-bearing EAC for 10 days, diluted with sterile 0.9% saline solution and then, vortex-mixed for 5 min. A 200 µL of the ascetic fluid after dilution was intramuscularly-injected into the right lower limb of male mice (22–25 g) for generation of Ehrlich solid tumor. Following 10 days inoculation, a palpable solid mass was detected.

##### Commencing the Biodistribution Study

The animals were divided into three groups (24 mice per group). The conscious mice were i.v. injected in the tail vein with 100 μL of ^99m^Tc-QRC solution (group A), ^99m^Tc-QRC-NOVs (group B) and ^99m^Tc-QRC-SeNOVs (group C) at a QRC dose equivalent to 10 mg/kg body weight [7]. Animals were euthanized after 0.08, 0.25, 0.5, 1, 2, 4, 6, and 24 h post injection.

##### Animal Handling and Dissection

A mixture of ketamine (80 mg/kg) and xylazine (10 mg/kg) was used to anesthetize the investigated mice (*n* = 3) at every time interval followed by weighing and dissection. Collection of blood samples was carried out through cardiac puncture and the entire organs (stomach, intestine, liver, spleen, heart, kidney, lung, brain, and bone) were isolated and cleaned by physiological saline. Regarding the tumorous muscle, the tumor sample was completely separated from the mouse leg, rinsed with physiological saline, then dried and weighed. The contralateral muscle was employed as a normal control for estimation of the uptake of the three radiolabeled formulations. The collected samples were introduced into pre-weighed plastic vessels wherein counting of the activity via an automatic scintillation γ-counter (scalar ratemeter SR-7, Nuclear Enterprises Ltd., Beenham, UK) was established. A standardized dose comprising similar injected quantity was concurrently counted and deemed as 100% activity. The uptake was described as % injected dose/g tissue (%ID/g) [33]. Muscles, bone, and blood were presumed to be 40, 10, and 7% of the whole-body weight, respectively.

##### Pharmacokinetic Data Analysis

The pharmacokinetics parameters of the three radiolabeled formulations were assessed utilizing Excel Add-Ins program, PK Solver software by non-compartmental analysis [25]. The average QRC radioactivity uptake (%ID/g) in blood and different tissues samples were graphed versus time (h) so that the maximal concentration of QRC uptake (C_max_) and the time to attain it (T_max_) were easily estimated. The area under the curve from 0 to 24 h (AUC_0–24_, h %ID/g), the area under the curve from 0 to infinity (AUC_0-∞_, h %ID/g), the time to reach half plasma concentration (T_1/2_, h), and the mean residence time (MRT, h) were also recorded.

##### Evaluation of Targeting Efficiency

For appraising the tumor-targeting efficacy of the tailored nano-cargo, the relative uptake efficiency (*RE*), concentration efficiency (*CE*), and drug-targeting efficiency (*DTE*) were computed utilizing the following equations [34]:

(3) $$RE=\frac{\;Tumor\;AUC_{\left(0-24\right)\;}after\;i.v.\;adminstraion\;of\;NOVs}{Tumor\;AUC_{\left(0-24\right)}\;after\;i.v.\;adminstraion\;of\;drug\;solution\;}\;$$

(4) $$CE=\frac{Tumor\;C_{max}\;of\;NOVs}{Tumor\;C_{max}\;of\;drug\;solution\;}\;$$

(5) $$DTE=\frac{Tumor\;AUC_{\left(0-24\right)\;}}{Blood\;AUC_{\left(0-24\right)}}\;$$

### 2.12. Statistical Analysis

All numerical data were presented as mean ± SD. Statistical analysis was executed utilizing one-way ANOVA followed by Tukey’s post hoc test through the computer software program SPSS 22 (SPSS, Chicago, IL, USA), where the value of *p* < 0.05 was regarded statistically significant.

## 3. Results and Discussion

### 3.1. Fabrication and Optimization of QRC-NOVs

Preliminary experimentations were executed for precise selection of the most adequate method for fabrication of QRC-NOVs. Additionally, preliminary trials were pursued to assign the appropriate drug solvent, free fatty acid, non-ionic surfactant, and sonication time. The thin film hydration method was carried out where QRC was integrated upon film preparation via being dissolved into the chosen organic solvent. Our target was the incorporation of 10 mg QRC into the NOVs dispersion. Thus, QRC solubility was screened in various organic solvents alongside the tested surfactants, free fatty acids, and cholesterol. Methanol, ethanol, acetone, and methylene chloride, in 7.5, 10, and 20 mL volumes, failed to dissolve the film forming constituents to initiate the film formation process, thereby mixtures of these solvents were tested. The solvent blend, which yielded a continuous and clear film was the mixture of methylene chloride and ethanol in a ratio of 2:1 with a final volume of 10 mL. Also, various free fatty acids as well as surfactants were examined, where oleic acid and Brij 35 were picked because they accomplished the smallest vesicle size and high EE% (data not shown). Concerning the sonication time, the NOVs were elaborated without sonication as well as sonicated for 10 or 20 min. The longer sonication produced small-sized vesicles meanwhile, the EE% values of the assembled NOVs were markedly lowered after 20 min sonication. Thus, the sonication time was limited to 10 min.

**Figure 1:**
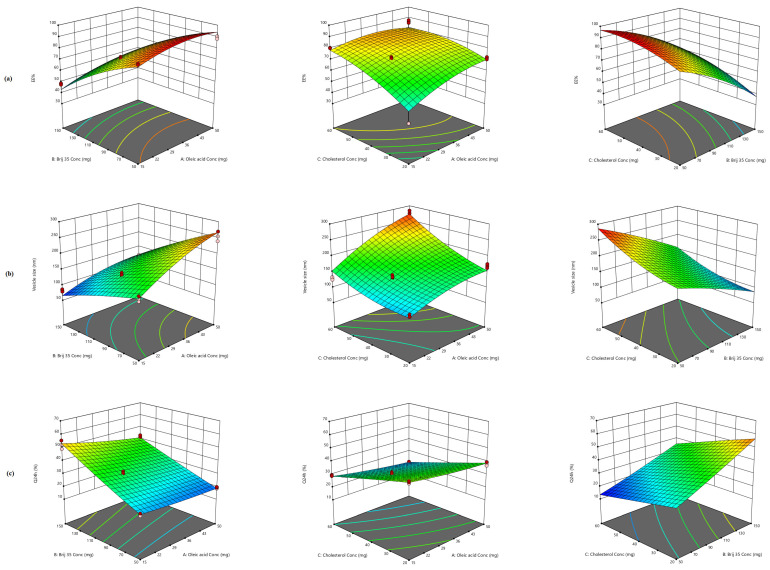
Response surface 3D plot for the effect of oleic acid (*X*_1_), Brij 35 (*X*_2_), and cholesterol (*X*_3_) concentrations on (**a**) EE% (*Y*_1_), (**b**) vesicle size (*Y*_2_), and (**c**) Q_24h_ (*Y*_3_).

Adopting Box–Behnken design, 15 runs with three checkpoints were generated for the formulation of QRC-NOVs, Table 2. The screened causal factors, at three different levels, were oleic acid (*X*_1_), Brij 35 (*X*_2_), and cholesterol (*X*_3_) concentrations. The tailored formulations were assessed for EE% (*Y*_1_), vesicle size (*Y*_2_), and Q_24h_ (*Y*_3_). The quadratic model was remarked as the best fit model for all the three dependent variables as presented in Table 3. The quadratic values of the measured responses together with their adequate precision, R^2^, SD, and % CV are summarized, Table 3. Furthermore, response 3D surface plots were constructed to depict the influences of the causal factors on the dependent variables, Figure 1.

### 3.2. QRC-NOVs Characterization

#### 3.2.1. Effect of Formulation Variables on EE%

Actually, high drug trapping within the vesicular assembly is essential to deliver an adequate quantity of the drug [23]. In the present study, the percentage of QRC retained by the NOVs formulations fluctuated between 35.21 ± 1.86 and 92.87 ± 2.56%, Table 2. Figure 1a illustrates the response surface plot for the combined impact of two independent variables on the EE% of QRC-NOVs at the middle level of the third variable. The EE% data were best-fitted to the quadratic model with F-value of 60.77, which was statistically significant (*p* < 0.0001) as displayed in Table 3. The regression equation encompassing the influence of the three independent variables on the EE% (*Y*_1_) in terms of coded values is represented by Equation (6).

(6) $$\begin{array}{c} EE\%=+78.44+5.23X_{1\;}-20.29X_{2\;}+9.31X_{3\;}+1.19\;X_{1\;}X_{2\;}-4.35\;X_{1}X_{3}\\ +2.70X_{2}X_{3}-4.36X_{1}^{2\;}-4.37X_{2}^{2}-4.29X_{3}^{2} \end{array}$$

The positive value preceding a factor in the regression equation specifies that the response increases with the factor and vice versa [35]. Equation (6) discloses a significant synergistic effect of oleic acid concentration (*X*_1_) on EE%, *p* < 0.0001. A plausible explanation for this successful QRC entrapment onto NOVs might be the robust interactions through van der Waals forces as well as hydrophobic attractions amongst the sparsely soluble drug and the lipophilic domain of the lipid bilayer warranting an entire amalgamation [36]. Additionally, the existence of oleic acid in the lipid bilayer could modulate the crystallization array as a result of its unsaturation and liquid trait conferring comparative imperfections in the bilayer chains, and thus maintaining the laden drug with avoidance of its expulsion [37]. Moreover, it was established that the presence of liquid phase, oleic acid, in the vesicular nano-cargo might endow an adequate space to accommodate the particles of the drug in the amorphous network of the system provoking an enriched EE% [38]. This finding is in harmony with that of Gabr et al. [39] who reported raised EE% of rosuvastatin from 63 to 92% with addition of oleic acid in the lipid domain upon formulation of hexagonal liquid crystalline nanodispersions presenting a privilege for higher drug loading capacity.

The impact of surfactant concentration (*X*_2_) was also investigated in our analysis. The negative coefficient before Brij 35 concentration (*X*_2_) indicates an inverse effect on the EE% of the primed NOVs (*p* < 0.0001). These results could be explained in the notion of the increased vesicular membrane fluidization established by the increment of the surfactant levels, which expedites leakage of the trapped drug [40]. In addition, the presence of high surfactant concentrations could lead to pores formation in the lipid bilayer and thereby, diminishing the EE% [41]. Furthermore, potential QRC solubilization by Brij 35, ensuing from its high HLB (16.9), could trigger drug diffusion in the external aqueous milieu during elaboration [35]. Comparable results were presented by Mahmoud et al. [42] who declared lower EE% of atorvastatin calcium-based nanovesicular systems comprising high levels of edge activators with analogous HLB values.

By further analysis of the response surfaces for the efficacy of encapsulation, it was obvious that cholesterol concentration (*X*_3_) exerted a positive influence on QRC EE%, *p* < 0.0001. This could be ascribed to the aptitude of cholesterol to augment membrane rigidity, stabilize the lipid bilayer chains, and amend the pattern of orientation ordering with a subsequent assemblage of less leaky vesicles that contributed to superior EE% [43]. This result is in a close agreement with that narrated by Abdelbary and El-Gendy [44] who developed gentamicin-loaded niosomes for controlled ophthalmic delivery.

#### 3.2.2. Effect of Formulation Variables on Vesicle Size and PDI

Since the aim of our work was the i.v. systemic delivery of QRC, small vesicle size was very eligible during the preparation of NOVs. The vesicle size of all NOVs formulations oscillated between 62.21 ± 9.15 and 274.47 ± 19.23 nm denoting a nanoscale range as profiled in Table 2. PDI values were employed to prognosticate the homogeneity of the assembled nanovesicles. A value of zero elucidates a monodispersed vesicular system, whilst a value of one indicates a system of high polydispersity [45]. PDI values of the tailored NOVs ranged from 0.112 to 0.417 manifesting confined size distribution and remarkable homogeneity. ANOVA test for the observed vesicle size data specified that the quadratic model was significant and fitting for the data. The quantitative impact of the three causal factors on the vesicle size of QRC-NOVs in coded values is donated by Equation (7).

(7) $$\begin{array}{ll} Vesicle\;size= & +169.62+42.95X_{1}-57.95X_{2}+42.06X_{3}-14.85X_{1}X_{2}\\ & +16.64X_{1}X_{3}-7.08X_{2}X_{3}-13.77X_{1}^{2}-4.80X_{2}^{2}+14.62X_{3}^{2} \end{array}$$

The inspected independent variables revealed a significant impact on the vesicle size among the various nanoformulations (*p* < 0.0001). It is worth noting that higher oleic acid concentrations (*X*_1_) were associated with a significant increase in the vesicle size of the prepared QRC-NOVs (*p* < 0.0001), Figure 1b. This relative vesicle size increase could be succumbed to the influence of raised viscosity attained with the increment of oleic acid concentration to higher levels. Also, the hydrophobic attractions as well as the molecular packing modulation as previously mentioned might be another reason for the manifested vesicle size growing [39]. Indeed, these findings are not surprising if they are interpreted in the light of EE% values where higher oleic acid content was coupled with greater QRC entrapped quantity within the vesicles, and hence a larger vesicle size was generated. Our results coincide with that claimed by Pinilla et al. [46] who reported increased nanovesicles size and ζ potential upon the addition of oleic acid during fabrication of freeze-dried liposomes encapsulating natural antimicrobials. On the other hand, Kelidari et al. [47] observed a decreased particle size concomitant with increased amount of oleic acid during preparation of spironolactone nanoparticles. These contradictory findings may be argued in terms of variances in the nature and lipid affinity of different drugs used.

Contrarily, the ANOVA results elicited a favorable antagonistic effect of Brij 35 concentration (*X*_2_) on the mean vesicle size (*p* < 0.0001). This finding could be explained on the basis of the decrement in the interfacial tension at higher Brij 35 level with consequent assembly of small-sized vesicles. On the other hand, a lower level of surfactant might be incompetent to enclose the overall vesicle perimeter; therefore, clustering of vesicles with reduced surface area could be prevailed [41]. El Menshawe et al. [35] shared similar results in their study on tailoring of fluvastatin sodium-loaded spanlastic nanovesicles. Moreover, the small vesicle size observed at high surfactant levels might be due to the development of mixed micelles rather than vesicles that possess a smaller size [42].

Similar to oleic acid, cholesterol concentration (*X*_3_) had a synergistic impact on the vesicle size of the prepared QRC-NOVs (*p* < 0.0001), Figure 1b. This could be accredited to the expansion of the lipid bilayers width accompanied by increased vesicles size upon increasing cholesterol concentration pursuant to its bulky structure [48]. These data are parallel to that established by a previous work [49].

#### 3.2.3. Effect of Formulation Variables on Q_24h_

The release profiles of QRC from the tailored NOVs as well as the control solution in PBS pH 7.4 are presented in figure (Figure S1a,b, Supplemental File). The % QRC released from the aqueous solution within 6 h was nearly 98.31 ± 1.56% whereas QRC-NOVs formulations delayed its release and the Q_24h_ ranged from 14.15 ± 2.86 to 59.66 ± 2.31%, as recorded in Table 2. These results elucidated the familiar reservoir impact of the vesicular systems that could denote a sustained release carrier for QRC [42]. The release profiles of QRC from various NOVs dispersions were distinctly biphasic processes where prompt release of the surface-resided drug was recognized over the early phase (first 2 h) escorted by an extended release pattern. For consummating the ANOVA presumption, the Box–Cox plot of Q_24h_ proposed square root response transformation with lambda = 0.5. The suggested model after transformation for Q_24h_ was polynomial quadratic model. The transformed regression equation correlating the response variation to the three causal factors in coded values was:

(8) $$\begin{array}{c} Sqrt\left(Q_{24h}\right)=+5.71-0.33X_{1}+1.15X_{2}-0.77X_{3}-0.11X_{1}X_{2}-0.084X_{1}X_{3}\\ -0.010X_{2}X_{3}+0.054X_{1}^{2}-0.066X_{2}^{2}-0.060X_{3}^{2} \end{array}$$

Our results disclosed a significant effect of the scrutinized terms on Q_24h_ of QRC-NOVs (*p* < 0.0001). A notable finding is that oleic acid concentration (*X*_1_) had an unfavorable effect on the Q_24h_ as revealed by the negative value in the quadratic equation, Figure 1c. This phenomenon could be accounted for increased QRC hydrophobicity due to the prominent hydrophobic interaction with the lipid bilayers retaining the drug [50]. Additionally, this might be due to the assembly of oleic acid micelles, at higher levels, that are considered to have a more sluggish influence on drug release than vesicles [51]. As described before in vesicle size assessment, the lowering of oleic acid concentration in the NOVs dispersions would generate vesicles with smaller size providing a higher surface area divulged to the release milieu and thus, ameliorating QRC release. Such findings are endorsed with an earlier literature report [38].

As regards to Brij 35 concentration (*X*_2_), it had a significant favorable effect on the Q_24h_ of QRC-NOVs (*p* < 0.0001). These findings can be clarified via improved solubility of the entrapped QRC in addition to enhanced fluidity of the lipid bilayers at higher surfactant concentration, which could give rise to snowballed permeability and release of the drug [52]. Controversially, at low surfactant concentration, abated QRC release was detected as the lipid membranes were more organized and less leaky, which hindered the release of the drug [41]. Moreover, another way to understand these findings is to take their vesicle size into account, where smaller vesicular size was attainable at higher Brij 35 levels. These observations are consistent with that assumed by El Menshawe et al. [53] who fabricated terbutaline sulfate-loaded bilosomes.

According to statistical inspection of the release data, cholesterol concentration (*X*_3_) had a negative impact on the Q_24h_ (*p* < 0.0001). Cholesterol could diminish the permeability or leakage of the entrapped drug by amending membrane fluidity [54]. Moreover, the presence of cholesterol in the lipid bilayer might restrict motion of the entrapped drug minimizing its efflux, and hence extended drug retention was distinguished [55].

Linear regression examination of the observed release data implied that QRC release from the majority of the tailored dispersions conformed to Higuchi kinetic equation proving a diffusion-controlled model. These findings are congruent with many previous reports on drug-laden vesicular systems [35,41,42]. Additionally, the mechanism of QRC release from NOVs was further inspected adopting Korsmeyer–Peppas model. The estimated *n* values among various NOVs dispersions extended between 0.61 and 0.78 denoting the non-Fickian drug diffusion (0.5 < *n* < 1) and establishing anomalous release pattern in which drug diffusion might be coupled with swelling of the lipid bilayers [38,56].

### 3.3. Fabrication Optimization and Analysis of the Box–Behnken Design

The Box–Behnken design was employed for modeling and examination of the empirical runs where it entails lesser experimentations than a full-factorial design [57]. Adequate precision was utilized to determine the signal to noise ratio for confirming the suitability of the model to navigate the design space [58]. As indicated in Table 3, a ratio more than four (the eligible value) was remarked in the three responses. In addition, the predicated R^2^ was computed as an estimate of the model fitness to prognosticate the value of a dependent variable [59,60]. The difference between values of the adjusted and predicted R^2^ should be within nearly 0.2 for being in a plausible agreement [61]. In the current work, it is notable that the values of the predicted R^2^ were in a close agreement with those of the adjusted R^2^ in all measured responses. The desirability constraints for the optimal formulation (maximal EE% and Q_24h_ with minimal vesicle size) were assessed with an overall desirability value of 0.634. The optimized dispersion was tailored using 15 mg oleic acid, 114.13 mg Brij 35, in addition to 41.83 mg cholesterol. The proposed formulation was fabricated and scrutinized and the residual between the prognosticated and observed responses was minor elucidating the optimization process validity. Different responses of the optimum formulation are denoted in Table 4. Thus, this formulation was designated for subsequent investigations.

### 3.4. Formulation and Characterization of QRC-SeNOVs

**Figure 2:**
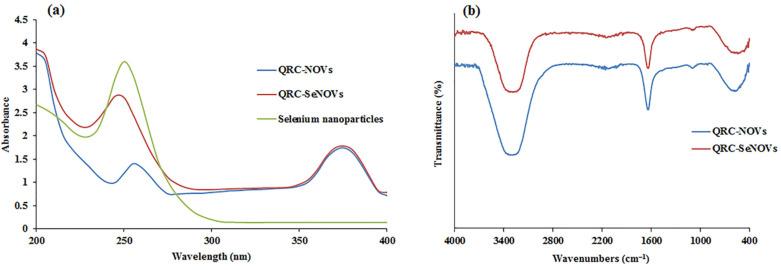
(**a**) UV–Vis spectra of QRC-NOVs, QRC-SeNOVs, and selenium nanoparticles; (**b**) FT-IR spectra of QRC-NOVs, and QRC-SeNOVs.

QRC-SeNOVs were fabricated based on the optimum QRC-NOVs by introducing sodium selenite and ascorbic acid into the system. Ascorbic acid elaborated as a good reductant that could interact with sodium selenite at a molar ratio of 5:1 [28]. According to this ratio, the vesicle size and PDI of QRC-SeNOVs were modified at different levels of sodium selenite within the system upon formulation. Beneath 0.28 mg/mL, the vesicle size of QRC-SeNOVs was raised with the increment of sodium selenite concentration, whereas beyond 0.28 mg/mL, the vesicle size and PDI were distinctly increased signifying the potential of selenium overload-triggered instability of NOVs. Thus, the employed concentration of sodium selenite for coating QRC-NOVs was 0.28 mg/mL, which conferred formation of a proper coating layer. The assembly of QRC-SeNOVs was initially investigated via UV-Vis, as well as FT-IR spectroscopy. As displayed in Figure 2a, bare selenium nanoparticles exhibited a UV absorbance peak at 250 nm emphasizing the surface-plasmon resonance of nanoselenium [62,63], whereas QRC-NOVs displayed the characteristic peaks of QRC at 256 and 374 nm, which is consistent with the literature data [64]. Further, QRC-SeNOVs manifested the drug absorbance peaks but with a maximal absorbance at 247.6 nm (i.e., close to the maximal absorbance of selenium nanoparticles). Such behavioral modulation of the observed optical spectra could account for selenium functionalization of QRC-NOVs. Taken together, the FT-IR spectrum of QRC-NOVs revealed a characteristic absorption peak at 3326 cm^−1^ for −OH stretching, which was shifted to a lower wavenumber (3307 cm^−1^) in case of QRC-SeNOVs, Figure 2b. The hydroxyl band shifting could denote a robust bonding interaction amongst hydroxyl groups of QRC-NOVs with atoms of selenium nanoparticles [65]. This observation is harmonious with previous arts reporting susceptibility of selenium to interaction with −OH groups [30,66]. Yu et al. [67] remarked an analogous blue-shift in the distinctive peak of hydroxyl group, at 3426.2 cm^−1^, in the tailored chitosan-selenium nanocomposites, which was comparatively lower than that of chitosan per se (3443.4 cm^−1^) deducing the −OH group and Se conjugation. Additionally, the impact of the amalgamated selenium nanoparticles on the dependent variables as EE% (*Y*_1_), vesicle size (*Y*_2_), and Q_24h_ (*Y*_3_) besides ζ potential was explored in our work. The EE% values for QRC-NOVs and QRC-SeNOVs were 67.21 ± 3.54 and 70.85 ± 2.42%, respectively, with a small elevation in case of QRC-SeNOVs, which might be due to structural upgrading of selenium nanoparticles that lowered the lipid bilayers leakage [28].

Expectedly, the average vesicle size of the untreated QRC-NOVs was assessed to be 107.29 ± 4.11 nm, which was increased to 129.16 ± 3.25 nm after selenium functionalization. This could be accredited to the emergent selenium precipitation that extended the dimensions of the formulated nanovesicles [68]. The ζ potential of QRC-NOVs was highly negative of −34.71 ± 4.61 mV, owing to the oleic acid carboxylic groups, which was decreased to −43.25 ± 2.74 mV after selenium plating. Such physicochemical changes confirmed precipitation of selenium onto the surface of QRC-NOVs upon reduction of Se^4+^ [28].

QRC-NOVs disclosed a slower release in PBS pH 7.4 with Q_24h_ of 43.26 ± 3.11%, while QRC-SeNOVs exhibited a much sluggish drug release relative to QRC-NOVs and the Q_24h_ was just 31.30 ± 2.25%. This pronounced sustained-release influence of QRC-SeNOVs could be inferred from selenium plating that triggered thickening of QRC-NOVs shell and reinforcing of the structure. Moreover, selenium decoration can reduce the outward drug diffusion from the lipid bilayers [69].

### 3.5. Transmission Electron Microscopy

**Figure 3:**
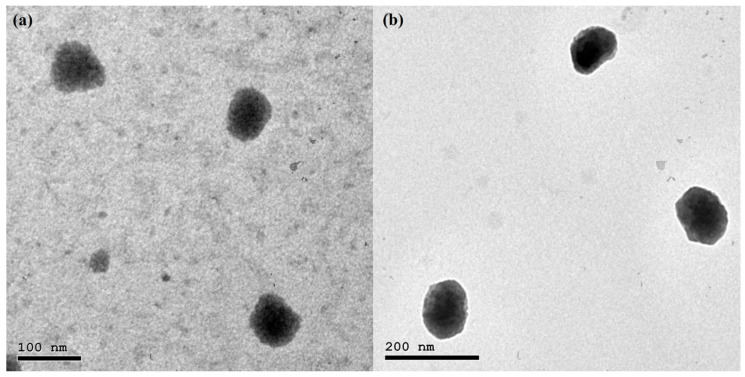
Transmission electron photomicrographs of (**a**) QRC-NOVs, and (**b**) QRC-SeNOVs.

TEM investigation is a valuable tool for exploring the shape, lamellarity, and size of nanovesicles [70]. TEM micrographs of the optimal QRC-NOVs and QRC-SeNOVs are clarified in Figure 3. The speculated vesicles were nanostructured, spherically shaped, unilamellar, non-aggregating vesicles with uniform size distribution. The photomicrograph of QRC-SeNOVs revealed a slight increment in vesicle size elucidating the adsorption of selenium coat, which appeared as a very thin layer surrounding the NOVs shell as shown in Figure 3b. Moreover, the average vesicles size attained via TEM analysis was in harmony with that measured by DLS.

### 3.6. Physical Stability Study

**Figure 4:**
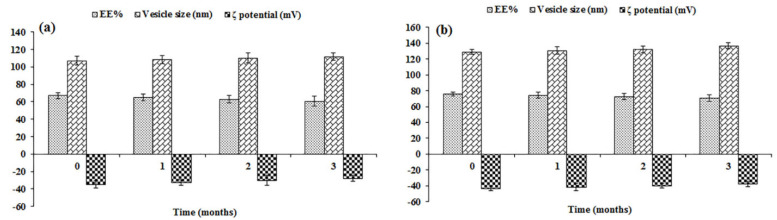
Effect of storage on EE%, vesicle size, and ζ potential of (**a**) QRC-NOVs, and (**b**) QRC-SeNOVs.

The EE%, vesicle size, and ζ potential of the optimized QRC-NOVs and QRC-SeNOVs were estimated after 30, 60, and 90 days as the main parameters to assess storage stability. Neither agglomeration nor abnormality was detected over the storage period. As demonstrated in Figure 4, the QRC EE%, vesicle size, and ζ potential exhibited insignificant change after three months of storage (*p* > 0.05), implying kinetic stability of the stored nanoformulations throughout this period. The perceived boosted stability emphasizes the prominence of oleic acid/Brij 35/cholestrol amalgamation. Also, such good stability could be assigned to the small vesicle size and narrow size distribution of the tailored nanovesicles. Additionally, QRC-NOVs and QRC-SeNOVs exhibited high ζ potential (>−25 mV), which could impart colloidal stability and hinder agglomeration of the vesicles [53]. Moreover, the remarkable stability of QRC-SeNOVs should underline the strong attractions among the terminal hydroxyl groups of QRC, oleic acid, Brij 35, and cholesterol with selenium nanoparticles, as discussed earlier, conferring a highly stable and well-dispersed spherical structure.

### 3.7. Cell Viability

**Figure 5:**
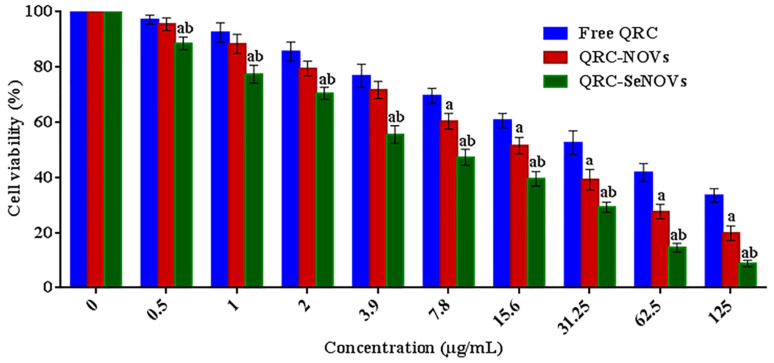
In vitro cytotoxicity of crude QRC, QRC-NOVs, and QRC-SeNOVs against RD cells via MTT assay after 48 h incubation. Data expressed as mean ± SD (*n* = 3). ^a^ *p* < 0.05 versus free QRC. ^b^ *p* < 0.05 versus QRC-NOVs.

In vitro cytotoxicity of crude QRC, QRC-NOVs, and QRC-SeNOVs against RD cells is represented in Figure 5. The three QRC formulations demonstrated concentration-dependent cytotoxic impacts. Though, the cytotoxicity of QRC-SeNOVs was superior to the activity exerted by both QRC-NOVs and free QRC. QRC-SeNOVs disclosed a significantly lower IC_50_ value (5.56 ± 0.78 μg/mL) on RD cells in comparison with QRC-NOVs (17.63 ± 1.82 μg/mL) and free QRC (38.71 ± 3.49 μg/mL), *p* < 0.05. Thus, SeNOVs could not only monitor QRC release rate but also ameliorate its cytotoxicity via synergism with selenium. As far as our work concerned, this is the first finding on the antitumor activity of QRC-SeNOVs.

### 3.8. Biodistribution and Pharmacokinetic Studies Using Radiolabeling Technique

#### 3.8.1. Radiolabeling of QRC, QRC-NOVs, and QRC-SeNOVs

**Figure 6:**
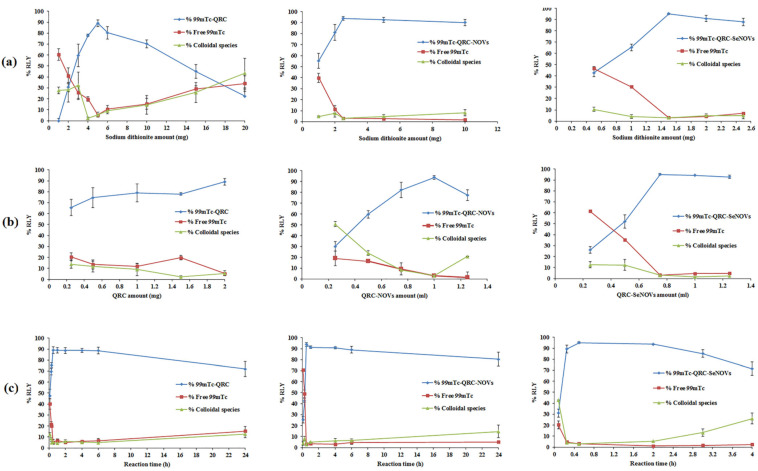
Effect of different conditions on the RLY of the ^99m^Tc-QRC complex: (**a**) sodium dithionite amount, (**b**) QRC amount, and (**c**) reaction time.

The radiolabeling with ^99m^Tc requires the reduction of ^99m^TcO_4_^−^ which has the oxidation state of 7^+^ with a tetrahydral configuration. In this oxidation state 7^+^, it is chemically nonreactive and cannot perform radiolabeling reactions. Thus, the reduction of ^99m^TcO_4_^−^ to a lower oxidation state is imminent. This reduction has been reported to be achieved using one of the following reducing agents; stannous chloride dihydrate (SnCl_2_·2H_2_O), sodium borohydride (NaBH_4_), and sodium dithionite (Na_2_S_2_O_4_). Recently, the radiolabeling of nanocarriers utilizing the reducing properties of sodium dithionite was evidently better than other reducing agents such as SnCl_2_·2H_2_O and NaBH_4_ as reported by Geskovski et al. [71]. So accordingly, the radiolabeling with ^99m^Tc was performed using the reducing properties of sodium dithionite. It is worth mentioning that the maximal RLY for ^99m^Tc-QRC, ^99m^Tc-QRC-NOVs, and ^99m^Tc-QRC-SeNOVs was obtained using 5, 2.5, and 1.5 mg of sodium dithionite, respectively, which was sufficient to reduce all the ^99m^TcO_4_^−^ in the reaction. Further increase in sodium dithionite concentration resulted in a decrease in the RLY, which might be due to colloidal precipitation [72]. As illustrated in Figure 6a, the lesser amount required for ^99m^Tc-QRC-SeNOVs could be attributed to the presence of a metal (selenium) on the surface of QRC-NOVs, which can efficiently chelate the reduced ^99m^Tc. Also, the highest RLY of ^99m^Tc-QRC, ^99m^Tc-QRC-NOVs and ^99m^Tc-QRC-SeNOVs was acquired utilizing 2, 1, and 0.75 mg of the substrate, respectively. The smaller substrate amount gave lower RLY, whereas a higher amount had insignificant effect as elucidated in Figure 6b. Additionally, time plays an important role in radiolabeling, hence determination of the suitable time for the reaction proceeding as well as the allowed time for the formed complex to be stable is very critical. For ^99m^Tc-QRC, ^99m^TC-QRC-NOVs, and ^99m^Tc-QRC-SeNOVs, the time required to proceed the reaction was 0.5 h for achieving the maximum RLY, Figure 6c. Moreover, it was observed that ^99m^Tc-QRC, and ^99m^Tc-QRC-NOVs exhibited good in vitro stability up to 6 h post labeling when kept at room temperature, whilst ^99m^Tc-QRC-SeNOVs was merely stable for 4 h then started to decline. Furthermore, the temperature triggered a negative impact on the RLY, where the optimum RLY was achieved at room temperature (25 ± 3 °C).

#### 3.8.2. Drug Biodistribution and Pharmacokinetic Studies

**Figure 7:**
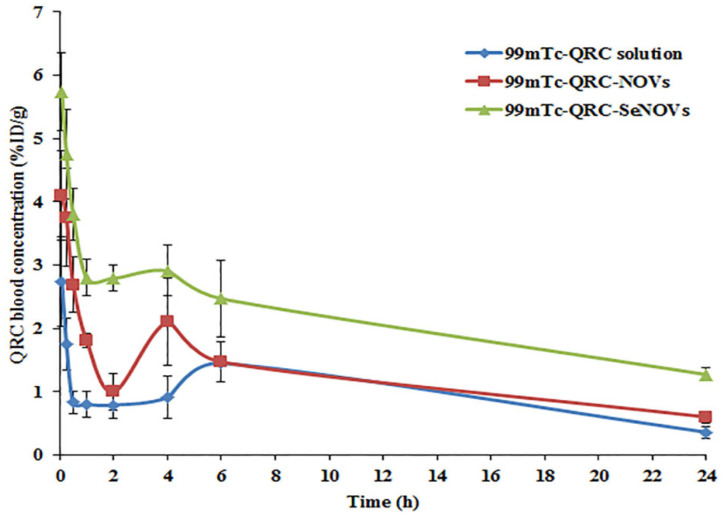
QRC blood concentration at various time intervals following i.v. administrations of ^99m^Tc-QRC solution, ^99m^Tc-QRC-NOVs, and ^99m^Tc-QRC-SeNOVs in Ehrlich tumor-bearing adult male Swiss albino mice, mean ± SD, *n* = 3.

In the present study, the radiolabeled formulations (^99m^Tc-QRC solution, ^99m^Tc-QRC-NOVs and ^99m^Tc-QRC-SeNOVs) were i.v. administered in Ehrlich tumor-bearing adult male Swiss albino mice. The radioactivity, estimated as %ID/g, was counted at several time intervals up to 24 h for various body organs or fluids, Table 5. Figure 7 denotes the mean QRC blood concentration in mice at various time intervals after i.v. administrations of the radiolabeled formulations. The QRC concentration in the blood could be rearranged in the following descending order; ^99m^Tc-QRC-SeNOVs > ^99m^Tc-QRC-NOVs > ^99m^Tc-QRC solution, at all-time points. These variations were verified to be statistically significant, *p* < 0.05. The pharmacokinetic variations among the radiolabeled dispersions were mathematically assessed according to the calculation of C_max_, T_max_, T_1/2_, MRT, AUC_0–24_, and AUC_0–∞_.

As summarized in Table 5, following i.v. administration of ^99m^Tc-QRC solution in mice, the maximum blood concentration (C_max_) attained was 2.74 ± 0.42% ID/g whereas the C_max_ of ^99m^Tc-QRC-NOVs, and ^99m^Tc-QRC-SeNOVs were 4.10 ± 0.72, and 5.74 ± 0.76% ID/g, respectively. The C_max_ of ^99m^Tc-QRC-NOVs, and ^99m^Tc-QRC-SeNOVs were significantly increased up to 1.5- and 2.09-fold, respectively, compared with ^99m^Tc-QRC solution, *p* ˂ 0.05. Also, the area under the concentration–time curve (AUC_0–∞_) of ^99m^Tc-QRC-NOVs, and ^99m^Tc-QRC-SeNOVs were 39.53 ± 5.42, and 51.64 ± 7.71 h %ID/g, respectively. The areas were approximately 1.32- and 1.72-fold greater than the AUC_0–∞_ of ^99m^Tc-QRC solution (30.06 ± 4.47 h %ID/g), respectively (*p* ˂ 0.05). Additionally, the mean residence time (MRT) of ^99m^Tc-QRC-NOVs, and ^99m^Tc-QRC-SeNOVs were 12.02 ± 2.64, and 21.30 ± 5.02 h, respectively, and they were higher compared to the MRT of ^99m^Tc-QRC solution (10.81 ± 2.24 h). Moreover, T_1/2_ of ^99m^Tc-QRC-NOVs was increased to nearly 8.11 ± 1.59 h, and that from the ^99m^Tc-QRC-SeNOVs was prolonged to 14.53 ± 3.52 h; for ^99m^Tc-QRC solution, T_1/2_ was 6.76 ± 1.13 h. A plausible explanation for the observed variations in the pharmacokinetic results might be due to the sustained-release trait of the engineered QRC-NOVs, and QRC-SeNOVs, which extended the residence time of QRC in the blood. In addition, selenium coating could trigger structural upgrading of NOVs, and supply QRC with a strikingly longer in vivo circulatory influence. Thus, it could be expected that QRC-SeNOVs would fortify QRC tumor accumulation through the EPR effect based on the pronged circulation [69]. Overtly, the results manifested a double-peak phenomenon in the concentration–time profiles following i.v. administration of the three investigated formulations. For ^99m^Tc-QRC solution, it is obvious that QRC is vulnerable to the enterohepatic recirculation in vivo [27]. Reabsorption of QRC, secreted with bile, would occur from the small intestine through the portal vein to the systemic circulation. For ^99m^Tc-QRC-NOVs, and ^99m^Tc-QRC-SeNOVs, such phenomenon could be owed to the enterohepatic recirculation, the nanovesicular size, as well as the capillary permeation. Intravenously-injected nano-cargos with a mean size beneath 7 µm are generally uptaken via the RES leading to their abundance within the RES organs [73,74]. Due to the spontaneous capillary permeability of these tissues, the distributed nano-cargos could diffuse to the interstitial spaces of such organs following i.v. administration, which warrant assembly of the nano-cargos in these tissues. Yet, smaller nano-cargos could infiltrate from the interstitial spaces and then, return to the systemic circulation triggering the double-peak pattern. Similarly, Jia et al. [75] reported the phenomenon of multi-peak upon biodistribution of intravenously-administered silybin-laded nanostructured lipid carriers.

Likewise, ^99m^Tc-QRC-NOVs, and ^99m^Tc-QRC-SeNOVs exhibited significant higher drug concentrations in the liver and spleen compared to ^99m^Tc-QRC solution (*p* ˂ 0.05). As mentioned above, this could be accounted for the potential metabolism and accumulation of the nanovesicular formulations via these organs with a sustained drug release from the tissues. Since release of the accumulated nano-cargos could occur from these organs, QRC would circulate for a prolonged duration in the blood, which should contribute to the efficiency accentuation.

The C_max_, and AUC_0–∞_ of QRC in the brain brought by ^99m^Tc-QRC-NOVs and ^99m^Tc-QRC-SeNOVs were approximately 1.33- and 3.11-fold, and about 1.55- and 2.68-fold higher, respectively compared to those of ^99m^Tc-QRC solution. The observed results could indicate the potential of the assembled vesicular nanocarriers to deliver QRC to the brain meanwhile the lipophilicity of QRC (*Log p* = 1.82) could rationalize the delivered fraction of the drug to the brain after i.v. administration of ^99m^Tc-QRC solution. Presumably, the size of nanovesicles is very critical for drug delivery to the brain since nanovesicles might be able to surpass the blood–brain barrier especially if their size is in the range of 70–345 nm [27].

Of note, ^99m^Tc-QRC divulged normal biological distribution in other body organs with higher radioactivity uptake in the case of ^99m^Tc-QRC-NOVs and ^99m^Tc-QRC-SeNOVs, which could be attributed to the permeation enhancing properties of the investigated nano-cargos, Table 5.

To evaluate the tumor targeting ability of a radiolabeled drug, the uptake of the target tissue (tumor, T) must be higher than non-target tissues (NT) [76]. High T/NT ratio indicates high selectivity of the radiolabeled compound towards the tumor sites and predicts a good imaging agent [77]. In this study, the uptake of contralateral normal muscle was considered as the non-target tissue along with the blood uptake. The T/NT ratios at 1 h following the i.v. administrations of ^99m^Tc-QRC solution, ^99m^Tc-QRC-NOVs, and ^99m^Tc-QRC-SeNOVs were 1.07 ± 0.11, 3.89 ± 0.54, and 5.23 ± 0.61, respectively, (Table S1, Supplemental File). As noted earlier, the significantly higher T/NT ratio of the developed QRC-SeNOVs could mark its tumor targeting potential, *p* < 0.05.

**Figure 8:**
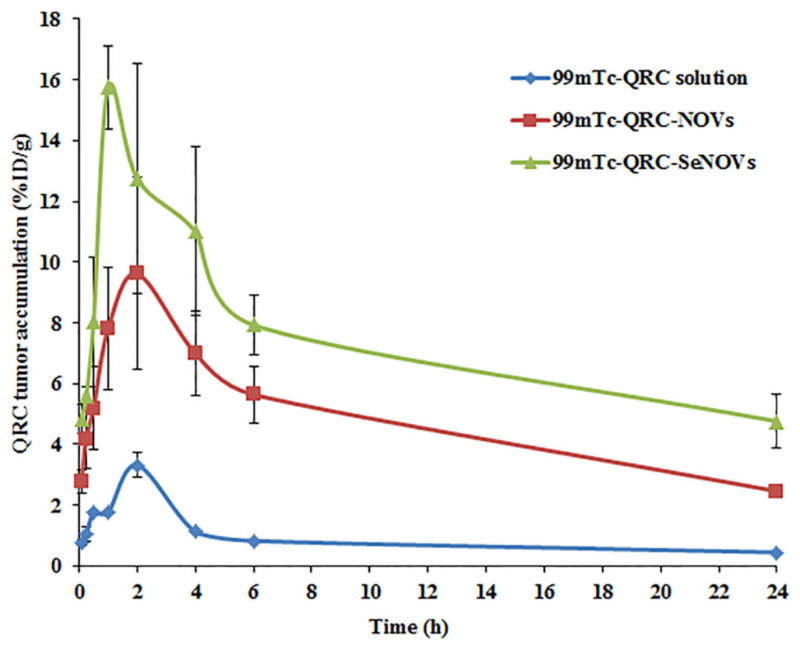
QRC tumor accumulation at various time intervals following i.v. administrations of ^99m^Tc-QRC solution, ^99m^Tc-QRC-NOVs, and ^99m^Tc-QRC-SeNOVs in Ehrlich tumor-bearing adult male Swiss albino mice, mean ± SD, *n* = 3.

The C_max_, AUC_0–∞_, and MRT values for the tumor uptake of QRC representing the i.v. ^99m^Tc-QRC solution, ^99m^Tc-QRC-NOVs and ^99m^Tc-QRC-SeNOVs were (3.32 ± 0.53, 9.61 ± 2.08, and 15.74 ± 2.22) %ID/g, (23.74 ± 8.52, 77.96 ± 11.68, and 134.49 ± 16.59) h %ID/g and (10.75 ± 3.53, 14.51 ± 2.97, and 26.69 ± 3.49) h, respectively, as shown in Table 5. The higher values accomplished with ^99m^Tc-QRC-SeNOVs were ascertained to be statistically significant (*p* < 0.05). In a parallel line, the T_max_ values for QRC tumor uptake after the i.v. administrations of ^99m^Tc-QRC solution, ^99m^Tc-QRC-NOVs and ^99m^Tc-QRC-SeNOVs were 2, 2 and 1 h, respectively, Figure 8. The significantly greater C_max_ and AUC_0–∞_ values alongside the shorter T_max_ following the administration of ^99m^Tc-QRC-SeNOVs could prove the preferential tumor targeting potential of the tailored nanovector. On the other hand, the more extended MRT might demonstrate its aptitude to monitor the drug release rate over an extended time period.

According to the AUC_0−24_ of the employed delivery systems in the muscle solid tumor and blood, the targeting parameters were calculated, Table 6. The relative efficiency (RE) of ^99m^Tc-QRC-NOVs, and ^99m^Tc-QRC-SeNOVs for the tumor accumulation was 3.28 ± 0.05, and 5.78 ± 0.06, respectively. Also, the concentration efficiency (CE), which can shed light on the biodistribution potential of a delivery system, was included in our analysis. The CE value of ^99m^Tc-QRC-SeNOVs was 1.64-fold significantly higher than the parallel mean value for ^99m^Tc-QRC-NOVs. As listed in Table 6, ^99m^Tc-QRC-SeNOVs evoked a 1.52- and 4.65-fold increase in the drug targeting efficiency (DTE) values as compared with ^99m^Tc-QRC-NOVs, and ^99m^Tc-QRC, respectively. Herein, the value of DTE > 3 could confirm excellent targeting and accumulation ability of ^99m^Tc-QRC-SeNOVs [34].

Summing up, the in vivo biodistribution study revealed boosted tumor accumulation of QRC-SeNOVs, which might be inferred from the following mechanisms: (i) the synergy of QRC-NOVs with selenium, a pleiotropic moiety, that possesses a greater selectivity for cancerous tissues and good aptness for passive tumor-targeting [78]; (ii) selenium functionalization could sustain QRC release conferring snowballed systemic exposure and circulation time; (iii) QRC-SeNOVs exhibited ameliorated pharmacokinetics (greater AUC, longer T_1/2_, and slower clearance) that provoked higher QRC distribution into the tumor; (iv) the tailored NOVs, oleic acid-enriched, could be deemed as nutraceuticals per se since oleic acid is a strategic moiety in cancer handicapping at certain doses [79]; and (v) the small size property of QRC-SeNOVs could offer a fruitful passive tumor-targeting capability via the EPR impact [80]. These findings are in line with Xie et al. [69] who reported reinforced pharmacokinetics, and anticancer activity of doxorubicin-loaded selenium-coated liposomes. Finally, the small size of the assembled QRC-SeNOVs permitted their extravasation with minimal risk of capillaries embolism, as well as easily endorsed sterilization through filtration, which will be beneficial for large scale fabrication once clinically available [81].

## 4. Conclusions

In this study, QRC-SeNOVs were successfully engineered via thin film hydration/active loading/in situ reduction method. Box–Behnken design was pursued for statistical optimization of the formulation variables. The optimum QRC-SeNOVs exhibited high EE%, small vesicle size, non-aggregating spherical structure, and prolonged in vitro release profile over 24 h. Moreover, QRC-SeNOVs provoked synergic cytotoxicity against RD cells. Additionally, the pharmacokinetic and biodistribution studies revealed higher AUC_0–∞_, extended T_1/2_ and MRT besides reinforced tumor uptake of ^99m^Tc-QRC-SeNOVs compared to ^99m^Tc-QRC-NOVs and ^99m^Tc-QRC solution. Our work presented a novel horizon for competent delivery of QRC via SeNOVs assembly, which might be constructive for the engineering of other anticancer medications. Nevertheless, additional preclinical investigations should be explored on appropriate animal models to warrant the superiority and safety of the tailored SeNOVs over the traditional QRC medications.

## Figures and Tables

**Table 1:** Box–Behnken design parameters, and constraints for QRC-NOVs.

Factor	Level of Variables		
	Low (−1)	Medium (0)	High (+1)
Independent variables			
*X*_1_: Oleic acid concentration (mg)	15	25	50
*X*_2_: Brij 35 concentration (mg)	50	100	150
*X*_3_: Cholesterol concentration (mg)	20	40	60
Dependent variables	Constraints		
*Y*_1_: EE%	Maximize		
*Y*_2_: Vesicle size (nm)	Minimize		
*Y*_3_: Q_24h_ (%)	Maximize		

EE%: entrapment efficiency percent; Q_24h_: accumulative % release after 24 h.

**Table 2:** QRC-NOVs experimental runs, causal factors, and observed responses based on the Box–Behnken design.

Run	Independent Variables			Dependent Variables			PDI
	*X*_1_ (mg)	*X*_2_ (mg)	*X*_3_ (mg)	*Y*_1_ (%)	*Y*_2_ (nm)	*Y*_3_ (%)	
R1 *	25	100	40	75.33 ± 2.82	146.37 ± 21.67	34.08 ± 1.18	0.291
R2	50	100	60	86.15 ± 7.29	274.47 ± 19.23	20.11 ± 2.74	0.173
R3	50	100	20	71.47 ± 2.75	169.21 ± 14.25	38.19 ± 1.39	0.382
R4*	25	100	40	76.34 ± 1.54	148.68 ± 28.37	34.22 ± 1.72	0.228
R5	15	50	40	88.31 ± 3.51	150.73 ± 12.85	22.54 ± 1.84	0.417
R6	50	150	40	52.63 ± 4.67	116.34 ± 17.36	41.36 ± 4.51	0.265
R7	50	50	40	89.67 ± 5.34	254.81 ± 23.55	19.12 ± 2.62	0.239
R8	25	150	20	35.21 ± 1.86	62.21 ± 9.15	59.66 ± 2.31	0.112
R9	15	100	60	80.36 ± 3.16	129.32 ± 20.73	29.23 ± 1.42	0.186
R10	25	50	60	92.87 ± 2.56	268.96 ± 19.48	14.15 ± 2.86	0.214
R11	15	150	40	48.23 ± 6.63	82.32 ± 7.78	52.26 ± 3.21	0.192
R12*	25	100	40	74.53 ± 2.25	150.99 ± 31.62	34.65 ± 2.83	0.153
R13	25	150	60	54.66 ± 1.34	139.18 ± 11.72	38.29 ± 1.64	0.371
R14	25	50	20	84.22 ± 5.12	163.67 ± 26.11	27.63 ± 1.23	0.332
R15	15	100	20	41.18 ± 3.73	108.86 ± 13.44	45.31 ± 1.46	0.127

*X*_1_: oleic acid concentration (mg); *X*_2_: Brij 35 concentration (mg); *X*_3_: cholesterol concentration (mg); *Y*_1_: entrapment efficiency percent; *Y*_2_: vesicle size (nm); *Y*_3_: accumulative release after 24 h (%); PDI: polydispersity index. Data are mean values (*n* = 3) ± SD. * Indicates the center point of the design.

**Table 3:** Regression analysis results for responses *Y*_1_, *Y*_2_, and *Y*_3_ for fitting data to different models.

Model	Adequate Precision	R^2^	Adjusted R^2^	Predicted R^2^	SD	% CV	*p* Value	Remarks
Response (*Y*_1_)								
Linear	26.15	0.8875	0.8788	0.8518	6.56	9.36	<0.0001	-
2FI	23.97	0.9112	0.8964	0.8650	6.06	8.65	<0.0001	-
Quadratic	24.83	0.9431	0.9276	0.8878	5.07	7.23	<0.0001	Suggested
Response (*Y*_2_)								
Linear	28.58	0.8969	0.8890	0.8581	21.08	13.36	<0.0001	-
2FI	29.77	0.9393	0.9291	0.8938	16.84	10.68	<0.0001	-
Quadratic	31.77	0.9644	0.9547	0.9300	13.47	8.54	<0.0001	Suggested
Response (*Y*_3_)								
Linear	72.27	0.9816	0.9801	0.9745	1.77	5.21	<0.0001	-
2FI	126.14	0.9962	0.9955	0.9940	0.84	2.47	<0.0001	-
Quadratic	121.46	0.9972	0.9964	0.9946	0.76	2.22	<0.0001	Suggested

*Y*_1_: entrapment efficiency percent; *Y*_2_: vesicle size (nm); *Y*_3_: accumulative release after 24 h (%); R^2^: coefficient of determination; SD: standard deviation; CV: coefficient of variation.

**Table 4:** Composition, predicted, and actual responses for the optimum QRC-NOVs formulation.

Optimum Formulation Composition (*X*_1_:*X*_2_:*X*_3_)	Response Variable	Predicted Value	Actual Value	Residual *
15.00:114.13:41.83	*Y*_1_	63.72	67.21	−3.49
	*Y*_2_	102.61	107.29	−4.68
	*Y*_3_	40.51	43.26	−2.75

*X*_1_: oleic acid concentration (mg); *X*_2_: Brij 35 concentration (mg); *X*_3_: cholesterol concentration (mg); *Y*_1_: entrapment efficiency percent; *Y*_2_: vesicle size (nm); *Y*_3_: accumulative release after 24 h (%). * Residual = Predicted − Actual.

**Table 5:** Pharmacokinetics parameters of QRC tissue distribution after i.v. administrations of ^99m^Tc-QRC solution, ^99m^Tc-QRC-NOVs, and ^99m^Tc-QRC-SeNOVs to Ehrlich tumor-bearing mice.

Organ/Tissue	C_max_	T_max_	K_elim_	T_1/2_	AUC_0–24_	AUC_0–∞_	MRT
^99m^Tc-QRC solution							
Blood	2.74 ± 0.42	0.08	0.1025 ± 0.0255	6.76 ± 1.13	26.91 ± 2.73	30.06 ± 4.47	10.81 ± 2.24
Liver	4.88 ± 0.87	2	0.0678 ± 0.0058	10.23 ± 1.03	68.33 ± 8.73	84.71 ± 17.37	13.61 ± 2.16
Kidney	6.00 ± 2.04	4	0.0614 ± 0.0087	11.29 ± 1.26	72.85 ± 9.82	98.25 ± 17.03	16.89 ± 2.24
Stomach	3.97 ± 0.47	4	0.0601 ± 0.0087	11.53 ± 1.97	58.95 ± 11.64	78.08 ± 16.37	16.19 ± 3.10
Intestine	3.84 ± 0.72	2	0.0775 ± 0.0118	8.95 ± 1.40	27.55 ± 3.66	32.06 ± 5.30	10.55 ± 1.34
Spleen	3.87 ± 0.42	4	0.0377 ± 0.0044	18.36 ± 2.26	64.85 ± 8.30	113.33 ± 26.94	27.34 ± 3.61
Lung	3.96 ± 0.73	4	0.1011 ± 0.0025	6.86 ± 1.17	38.08 ± 6.68	42.53 ± 7.28	9.87 ± 1.62
Heart	2.05 ± 0.31	2	0.0583 ± 0.0094	11.90 ± 1.69	19.89 ± 2.54	27.95 ± 4.37	17.73 ± 2.82
Bone	2.77 ± 0.45	2	0.0719 ± 0.0074	9.64 ± 2.12	21.85 ± 4.51	26.10 ± 5.32	12.14 ± 3.94
Brain	0.75 ± 0.08	4	0.1505 ± 0.0299	4.60 ± 0.34	9.85 ± 1.32	10.11 ± 1.16	6.44 ± 0.93
Muscle	2.98 ± 0.55	2	0.1031 ± 0.0088	6.72 ± 1.27	17.44 ± 2.31	20.97 ± 3.62	9.80 ± 2.06
Tumor	3.32 ± 0.53	2	0.0853 ± 0.0075	8.12 ± 1.59	18.70 ± 5.82	23.74 ± 8.52	10.75 ± 3.53
^99m^Tc-QCR-NOVs							
Blood	4.10 ± 0.72 ^a^	0.08	0.0854 ± 0.0089 ^a^	8.11 ± 1.59 ^a^	29.05 ± 3.55 ^a^	39.53 ± 5.42 ^a^	12.02 ± 2.64 ^a^
Liver	14.99 ±2.19 ^a^	4 ^a^	0.1002 ± 0.0356 ^a^	6.92 ± 1.12 ^a^	204.49 ± 27.88 ^a^	224.55 ± 39.82 ^a^	8.97 ± 1.04 ^a^
Kidney	13.45 ± 2.75 ^a^	4	0.0605 ± 0.0093	11.46 ± 2.55	132.61 ± 22.13 ^a^	177.43 ± 36.45 ^a^	16.01 ± 3.12
Stomach	4.65 ± 1.03 ^a^	4	0.0639 ± 0.0124	10.85 ± 2.27	69.32 ± 11.37 ^a^	88.88 ± 10.25 ^a^	14.90 ± 2.64 ^a^
Intestine	3.55 ± 0.46	2	0.0585 ± 0.0098 ^a^	11.84 ± 2.41 ^a^	48.20 ± 6.96 ^a^	64.42 ± 9.14 ^a^	16.33 ± 2.02 ^a^
Spleen	12.58 ± 1.38 ^a^	4	0.0859 ± 0.0089 ^a^	8.07 ± 1.65 ^a^	133.18 ± 26.52 ^a^	154.48 ± 32.62 ^a^	10.89 ± 1.35 ^a^
Lung	10.87 ± 2.03 ^a^	0.25 ^a^	0.0885 ± 0.0138 ^a^	7.83 ± 1.26 ^a^	61.27 ± 10.49 ^a^	70.87 ± 12.21 ^a^	10.21 ± 1.41
Heart	2.23 ± 0.64	0.5 ^a^	0.0415 ± 0.0065 ^a^	16.72 ± 2.77 ^a^	14.06 ± 1.83 ^a^	22.50 ± 3.26 ^a^	23.22 ± 4.13 ^a^
Bone	1.99 ± 0.31 ^a^	1 ^a^	0.0614 ± 0.0092 ^a^	11.29 ± 1.65 ^a^	30.81 ± 4.06 ^a^	39.77 ± 7.29 ^a^	15.13 ± 2.72 ^a^
Brain	1.00 ± 0.19 ^a^	1 ^a^	0.0574 ± 0.0126 ^a^	12.08 ± 1.65 ^a^	11.32 ± 2.13 ^a^	15.68 ± 2.69 ^a^	17.45 ± 3.14 ^a^
Muscle	3.01 ± 0.64	2	0.0769 ± 0.0076 ^a^	9.01 ± 2.76 ^a^	28.12 ± 3.66 ^a^	37.09 ± 6.64 ^a^	12.42 ± 4.78 ^a^
Tumor	9.61 ± 2.08 ^a^	2	0.0635 ± 0.0508 ^a^	10.91 ± 1.59 ^a^	61.40 ± 9.74 ^a^	77.96 ± 11.68 ^a^	14.51 ± 2.97 ^a^
^99m^Tc-QRC-SeNOVs							
Blood	5.74 ± 0.76 ^a,b^	0.08	0.0477 ± 0.0069 ^a,b^	14.53 ± 3.52 ^a,b^	33.63 ± 4.64 ^a,b^	51.64 ± 7.71 ^a,b^	21.30 ± 5.02 ^a,b^
Liver	16.01 ± 2.23 ^a,b^	4 ^a^	0.0585 ± 0.0093 ^a,b^	11.85 ± 2.63 ^a,b^	265.75 ± 21.72 ^a,b^	351.89 ± 28.29 ^a,b^	16.20 ± 2.82 ^a,b^
Kidney	15.32 ± 2.42 ^a,b^	4	0.0317 ± 0.0064 ^a,b^	21.87 ± 3.11 ^a,b^	178.03 ± 19.62 ^a,b^	355.71 ± 37.55 ^a,b^	32.68 ± 5.52 ^a,b^
Stomach	6.20 ± 1.37 ^a,b^	4	0.0506 ± 0.0065 ^a,b^	13.71 ± 2.91 ^a,b^	94.42 ± 13.82 ^a,b^	136.94 ± 23.12 ^a,b^	19.68 ± 2.47 ^a,b^
Intestine	5.21 ± 1.06 ^a,b^	4 ^a,b^	0.0576 ± 0.0115 ^a^	12.03 ± 2.33 ^a^	63.57 ± 9.11 ^a,b^	87 ± 13.21 ^a,b^	17.16 ± 3.05 ^a^
Spleen	13.17 ± 2.62 ^a^	4	0.0793 ± 0.0169 ^a^	8.74 ± 1.23 ^a^	167.83 ± 17.18 ^a,b^	198.58 ± 24.82 ^a,b^	11.77 ± 2.77 ^a^
Lung	13.91 ± 2.26 ^a,b^	0.25 ^a^	0.0763 ± 0.0115 ^a,b^	9.08 ± 0.82 ^a,b^	95.68 ± 8.43 ^a,b^	114.68 ± 16.89 ^a,b^	11.87 ± 1.17 ^a,b^
Heart	2.99 ± 0.45	1 ^a,b^	0.0543 ± 0.0132 ^b^	12.75 ± 2.21 ^b^	23.29 ± 3.24 ^a,b^	31.94 ± 7.04 ^a,b^	17.06 ± 4.39 ^b^
Bone	3.01 ± 0.74 ^b^	1 ^a^	0.0515 ± 0.0075 ^a,b^	13.45 ± 1.35 ^a,b^	35.92 ± 6.51 ^a,b^	50.09 ± 14.21 ^a,b^	18.09 ± 2.93 ^a,b^
Brain	2.33 ± 0.46 ^a,b^	1 ^a^	0.0628 ± 0.0142 ^a,b^	11.04 ± 2.85 ^a,b^	19.93 ± 3.14 ^a,b^	27.10 ± 4.46 ^a,b^	16.56 ± 2.45 ^a^
Muscle	3.67 ± 0.41 ^a,b^	2	0.0507 ± 0.0073 ^a,b^	13.67 ± 1.51 ^a,b^	40.83 ± 5.73 ^a,b^	60.93 ± 7.66 ^a,b^	20.52 ± 2.98 ^a,b^
Tumor	15.74 ± 2.22 ^a,b^	1 ^a,b^	0.0365 ± 0.0061 ^a,b^	18.97 ± 2.62 ^a,b^	108.11 ± 11.74 ^a,b^	134.49 ± 16.59 ^a,b^	26.69 ± 3.49 ^a,b^

^99m^Tc: technetium-99m; QRC: quercetin; NOVs: novasomes; SeNOVs: selenium-plated novasomes; C_max_: maximum drug concentration in plasma; T_max_: time to reach C_max_; K_elim_: elimination rate constant; T_1/2_: terminal half-life; AUC_0–24_: area under plasma concentration–time curve from 0 to 24 h; AUC_0–∞_: total area under plasma concentration–time curve; MRT: mean residence time. Each value is the mean ± SD of three separate determinations. Using one-way ANOVA followed by Tukey post hoc test. ^a^ *p* < 0.05 versus ^99m^Tc-QRC solution. ^b^ *p* < 0.05 versus ^99m^Tc-QRC-NOVs.

**Table 6:** Tumor specific targeting parameters after i.v. administrations of ^99m^Tc-QRC solution, ^99m^Tc-QRC-NOVs, and ^99m^Tc-QRC-SeNOVs to Ehrlich tumor-bearing mice.

Parameter	^99m^Tc-QRC Solution	^99m^Tc-QRC-NOVs	^99m^Tc-QRC-SeNOVs
RE	-	3.28 ± 0.05	5.78 ± 0.06 ^b^
CE	-	2.89 ± 0.11	4.74 ± 0.09 ^b^
DTE	0.69 ± 0.04	2.11 ± 0.13 ^a^	3.21 ± 0.08 ^a,b^

^99m^Tc: technetium-99m; QRC: quercetin; NOVs: novasomes; SeNOVs: selenium-plated novasomes; RE: relative uptake efficiency; CE: concentration efficiency; DTE: drug-targeting efficiency. Listed data are mean values ± SD (*n* = 3). Using one-way ANOVA followed by Tukey post hoc test. ^a^ *p* < 0.05 versus 99mTc-QRC solution. ^b^ *p* < 0.05 versus 99mTc-QRC-NOVs.

## Data Availability

All processed data in this work are incorporated into the article.

## Supplementary Materials

The following supporting information can be downloaded at: https://www.mdpi.com/article/10.3390/pharmaceutics14040875/s1, Figure S1. In vitro release profiles of QRC from drug solution and different NOVs runs: (a) R1–R8 and (b) R9–R15. Table S1. QRC Biodistribution after i.v. administrations of ^99m^Tc-QRC solution, ^99m^Tc-QRC-NOVs, and ^99m^Tc-QRC-SeNOVs to Ehrlich tumor-bearing mice at various time intervals.
