# Supplementary material for: Tailoring of Selenium-Plated Novasomes for Fine-Tuning Pharmacokinetic and Tumor Uptake of Quercetin: In Vitro Optimization and In Vivo Radiobiodistribution Assessment in Ehrlich Tumor-Bearing Mice
Source: Pharmaceutics. 2022 Apr 16;14(4):875. doi: 10.3390/pharmaceutics14040875 (PMC9032182; doi:10.3390/pharmaceutics14040875)
Supplement: Supplementary file 1 [file pharmaceutics-14-00875-s001.zip › pharmaceutics-1665252-supplementary.pdf]

pharmaceutics

MDPI

Supplementary Materials: Tailoring of Selenium-Plated Novasomes for Fine-Tuning Pharmacokinetic and Tumor Uptake of Quercetin: In Vitro Optimization and In Vivo Radiobiodistribution Assessment in Ehrlich Tumor-Bearing Mice

Heba M. Aboud, Amal K. Hussein, Abdallah Z. Zayan, Tarek Saad Makram, Mona O. Sarhan and Dina M. El-Sharawy

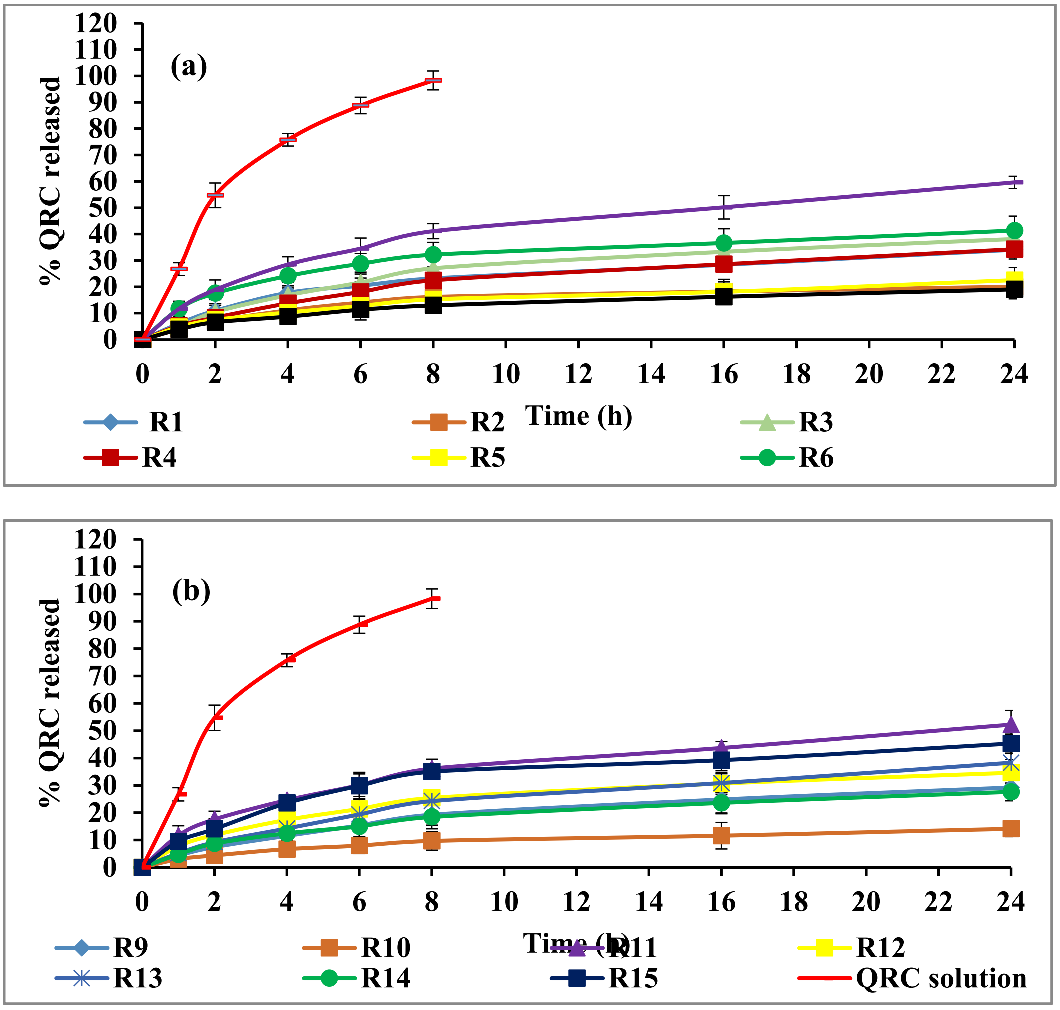

Figure S1. In vitro release profiles of QRC from drug solution and different NOVs runs: (**a**) R1–R8 and (**b**) R9–R15.

Table S1. QRC Biodistribution after i.v. administrations of ^{99m}Tc-QRC solution, ^{99m}Tc-QRC-NOVs, and ^{99m}Tc-QRC-SeNOVs to Ehrlich tumor-bearing mice at various time intervals.

**Organ/Tissue**	**Time (h)**							
	**0.08**	**0.25**	**0.5**	**1**	**2**	**4**	**6**	**24**
				^{99m}Tc-QRC solution				
blood	2.74 ± 0.71	1.75 ± 0.41	0.83 ± 0.17	0.80 ± 0.21	0.79 ± 0.22	0.91 ± 0.34	1.45 ± 0.05	0.36 ± 0.09
Muscle	0.37 ± 0.05	1.01 ± 0.18	1.36 ± 0.08	1.66 ± 0.36	2.98 ± 0.73	1.09 ± 0.04	0.66 ± 0.08	0.24 ± 0.01
Tumor	0.78 ± 0.11	1.05 ± 0.26	1.75 ± 0.09	1.77 ± 0.04	3.32 ± 0.42	1.13 ± 0.03	0.83 ± 0.01	0.45 ± 0.02
T/NT	2.11 ± 0.16	1.04 ± 0.08	1.29 ± 0.13	1.07 ± 0.11	1.11 ± 0.09	1.04 ± 0.05	1.26 ± 0.13	1.88 ± 0.21
T/blood	0.28 ± 0.02	0.60 ± 0.06	2.11 ± 0.12	2.21 ± 0.22	4.20 ± 0.28	1.24 ± 0.16	0.57 ± 0.03	1.25 ± 0.14
				^{99m}Tc-QRC-NOVs				
Blood	4.10 ± 0.71[a]	3.75 ± 0.77[a]	2.69 ± 0.44[a]	1.30 ± 0.11[a]	1.00 ± 0.29[a]	2.10 ± 0.69[a]	1.47 ± 0.31	0.60 ± 0.09[a]
Muscle	0.88 ± 0.11[a]	1.30 ± 0.12[a]	1.61 ± 0.09[a]	2.01 ± 0.31[a]	3.01 ± 0.97	1.67 ± 0.15[a]	1.09 ± 0.15[a]	0.76 ± 0.01[a]
Tumor	2.78 ± 0.39[a]	4.19 ± 0.97[a]	5.19 ± 1.37[a]	7.81 ± 2.03[a]	9.61 ± 3.16[a]	7.00 ±1.39[a]	5.64 ± 0.94[a]	2.46 ± 0.07[a]
T/NT	3.16 ± 0.63[a]	3.22 ± 0.71[a]	3.22 ± 0.42[a]	3.89 ± 0.54[a]	3.19 ± 0.62[a]	4.19 ± 0.31[a]	5.17 ± 0.83[a]	3.24 ± 0.52[a]
T/blood	0.68 ± 0.15[a]	1.12 ± 0.11[a]	1.93 ± 0.16	6.01 ± 0.87[a]	9.61 ± 1.23[a]	3.33 ± 0.58[a]	3.84 ± 0.63[a]	4.10 ± 0.81[a]
				^{99m}Tc-QRC-SeNOVs				
Blood	5.74 ± 0.61[a,b]	4.75 ± 0.70[a,b]	3.80 ± 0.41[a,b]	2.80 ± 0.29[a,b]	2.79 ± 0.20[a,b]	2.91 ± 0.40[a,b]	2.47 ± 0.60[a,b]	1.27 ± 0.09[a,b]
Muscle	0.97 ± 0.06[a]	1.52 ± 0.39[a,b]	2.60 ± 0.27[a,b]	3.01 ± 0.97[a,b]	3.67 ± 0.47[a,b]	2.00 ± 0.15[a,b]	1.87 ± 0.12[a,b]	0.99 ± 0.02[a,b]
Tumor	4.83 ± 0.47[a,b]	5.61 ± 0.27[a,b]	8.03 ± 2.14[a,b]	15.74 ± 1.36[a,b]	12.74 ± 3.79[a,b]	11.00 ± 2.78[a,b]	7.93 ± 0.97[a,b]	4.76 ± 0.89[a,b]
T/NT	4.98 ± 0.76[a,b]	3.69 ± 0.72[a]	3.09 ± 0.41[a]	5.23 ± 0.61[a,b]	3.47 ± 0.55[a]	5.50 ± 0.92[a,b]	4.24 ± 0.66[a,b]	4.81 ± 0.83[a,b]
T/blood	0.84 ± 0.26[a,b]	1.18 ± 0.35[a]	2.11 ± 0.31	5.62 ± 0.68[a]	4.57 ± 0.84[a,b]	3.78 ± 0.67[a]	3.21 ± 0.56[a,b]	3.75 ± 0.49[a]

^{99m}Tc: technetium-99m; QRC: quercetin; NOVs: novasomes; SeNOVs: selenium-plated novasomes; T/NT (target/non-target ratio) = tumor muscle/normal muscle.Listed data are mean values ± SD (n = 3).Using one-way ANOVA followed by Tukey post-hoc test. [a] $p < 0.05$ versus 99mTc-QRC solution. [b] $p < 0.05$ versus 99mTc-QRC-NOVs.
